# U-Net-based computed tomography quantification of viral pneumonia can predict fibrotic interstitial lung abnormalities at 3-month follow-up

**DOI:** 10.3389/fmed.2024.1435337

**Published:** 2024-09-30

**Authors:** Zhoumeng Ying, Zhenchen Zhu, Ge Hu, Zhengsong Pan, Weixiong Tan, Wei Han, Zifeng Wu, Zhen Zhou, Jinhua Wang, Wei Song, Lan Song, Zhengyu Jin

**Affiliations:** ^1^Department of Radiology, State Key Laboratory of Complex Severe and Rare Diseases, Peking Union Medical College Hospital, Chinese Academy of Medical Sciences and Peking Union Medical College, Beijing, China; ^2^4+4 Medical Doctor Program, Chinese Academy of Medical Sciences & Peking Union Medical College, Beijing, China; ^3^Theranostics and Translational Research Center, National Infrastructures for Translational Medicine, Institute of Clinical Medicine, Peking Union Medical College Hospital, Chinese Academy of Medical Sciences and Peking Union Medical College, Beijing, China; ^4^Deepwise AI Lab, Beijing Deepwise and League of PhD Technology Co. Ltd., Beijing, China; ^5^Department of Epidemiology and Health Statistics, Institute of Basic Medicine Sciences, School of Basic Medicine, Chinese Academy of Medical Sciences and Peking Union Medical College, Beijing, China

**Keywords:** post-acute COVID-19 syndrome, pulmonary fibrosis, lung diseases, interstitial, artificial intelligence, multidetector computed tomography

## Abstract

**Background:**

Given the high prevalence of fibrotic interstitial lung abnormalities (ILAs) post-COVID-19, this study aims to evaluate the effectiveness of quantitative CT features in predicting fibrotic ILAs at 3-month follow-up.

**Methods:**

This retrospective study utilized cohorts from distinct clinical settings: the training dataset comprised individuals presenting at the fever clinic and emergency department, while the validation dataset included patients hospitalized with COVID-19 pneumonia. They were classified into fibrotic group and nonfibrotic group based on whether the fibrotic ILAs were present at follow-up. A U-Net-based AI tool was used for quantification of both pneumonia lesions and pulmonary blood volumes. Receiver operating characteristic (ROC) curve analysis and multivariate analysis were used to assess their predictive abilities for fibrotic ILAs.

**Results:**

Among the training dataset, 122 patients (mean age of 68 years ±16 [standard deviation], 73 men), 55.74% showed fibrotic ILAs at 3-month follow-up. The multivariate analysis identified the pneumonia volume [PV, odd ratio (OR) 3.28, 95% confidence interval (CI): 1.20–9.31, *p* = 0.02], consolidation volume (CV, OR 3.77, 95% CI: 1.37–10.75, *p* = 0.01), ground-glass opacity volume (GV, OR 3.38, 95% CI: 1.26–9.38, *p* = 0.02), pneumonia mass (PM, OR 3.58, 95% CI: 1.28–10.46, *p* = 0.02), and the CT score (OR 12.06, 95% CI: 3.15–58.89, *p* < 0.001) as independent predictors of fibrotic ILAs, and all quantitative parameters were as effective as CT score (all *p* > 0.05). And the area under the curve (AUC) values were PV (0.79), GV (0.78), PM (0.79), CV (0.80), and the CT score (0.77). The validation dataset, comprising 45 patients (mean age 67.29 ± 14.29 years, 25 males) with 57.78% showing fibrotic ILAs at follow-up, confirmed the predictive validity of these parameters with AUC values for PV (0.86), CV (0.90), GV (0.83), PM (0.88), and the CT score (0.85). Additionally, the percentage of blood volume in vessels <5mm^2^ relative to the total pulmonary blood volume (BV5%) was significantly lower in patients with fibrotic ILAs (*p* = 0.048) compared to those without.

**Conclusion:**

U-Net based quantification of pneumonia lesion and BV5% on baseline CT scan has the potential to predict fibrotic ILAs at follow-up in COVID-19 patients.

## 1 Introduction

Pulmonary fibrosis represents the final stage in the progression of various interstitial lung diseases, resulted from over 200 contributing factors, including viral infections, or environmental toxins (1, 2). Notably, a meta-analysis has indicated that viral infections can increase the risk of Idiopathic Pulmonary Fibrosis (IPF) (3). Fibrotic changes in CT, which were referred to fibrotic interstitial lung abnormalities (ILAs) by the Fleischner Society Glossary, are considered as crucial precursors to idiopathic pulmonary fibrosis (4, 5). However, in a two-year follow-up study, 23% of COVID-19 survivors exhibited persistent fibrotic ILAs, with symptoms like dry cough, breathlessness, and impaired lung function, Markedly reducing their quality of life (6). Considering the widespread transmission of COVID-19 (7), the emergence of fibrotic ILAs poses a substantial health challenge. Despite the lack of standardized treatment, early intervention with antifibrotic medications (8) and steroids (9) has shown promise in preventing the onset and progression of fibrotic ILAs. Consequently, accurately predicting the occurrence of fibrotic ILAs during the acute phase of infection is crucial, as it allows for the early adoption of active treatment approaches, potentially averting the development of fibrotic ILAs (10).

Risk factors of fibrotic ILAs include patient characteristics like advanced age and smoking, and clinical indicators such as high CRP and IL-6 levels (11). Crucially, the initial imaging, particularly the visual CT score, serves as a significant predictor of fibrotic ILAs, highlighting the vital role of early radiological findings in forecasting the development of fibrotic ILAs (12–14).

The assessment of chest CT images, traditionally performed by radiologists, can be influenced by subjective bias (15). Deep learning algorithms offer a more precise and consistent method for the quantitative analysis of abnormal lung findings, potentially improving the identification of pneumonia-related lesions in COVID-19 patients (16–18). Preliminary research has demonstrated that quantitative analysis of early COVID-19 CT scans, including pneumonia volume (PV), ground-glass opacity volume (GV), consolidation volume (CV), and the percentage of blood volume contained in vessels with a cross-sectional area less than 5 mm^2^ (BV5%) can be predictive of patient outcomes (16–20). Nonetheless, research that employs quantitative analysis of initial COVID-19 chest CT to predict the development of fibrotic ILAs remains scarce (13).

In this study, we aimed to examine the utility of quantitative CT features, as compared with traditional radiologist-defined CT features in predicting the development of fibrotic ILAs in COVID-19 pneumonia patients after at 3-month follow-up.

## 2 Methods

The study was approved by the institutional ethic committee of our hospital (approval number: K2926) and individual consent for this retrospective analysis was waived.

### 2.1 Patient population and study design

**Figure 1:**
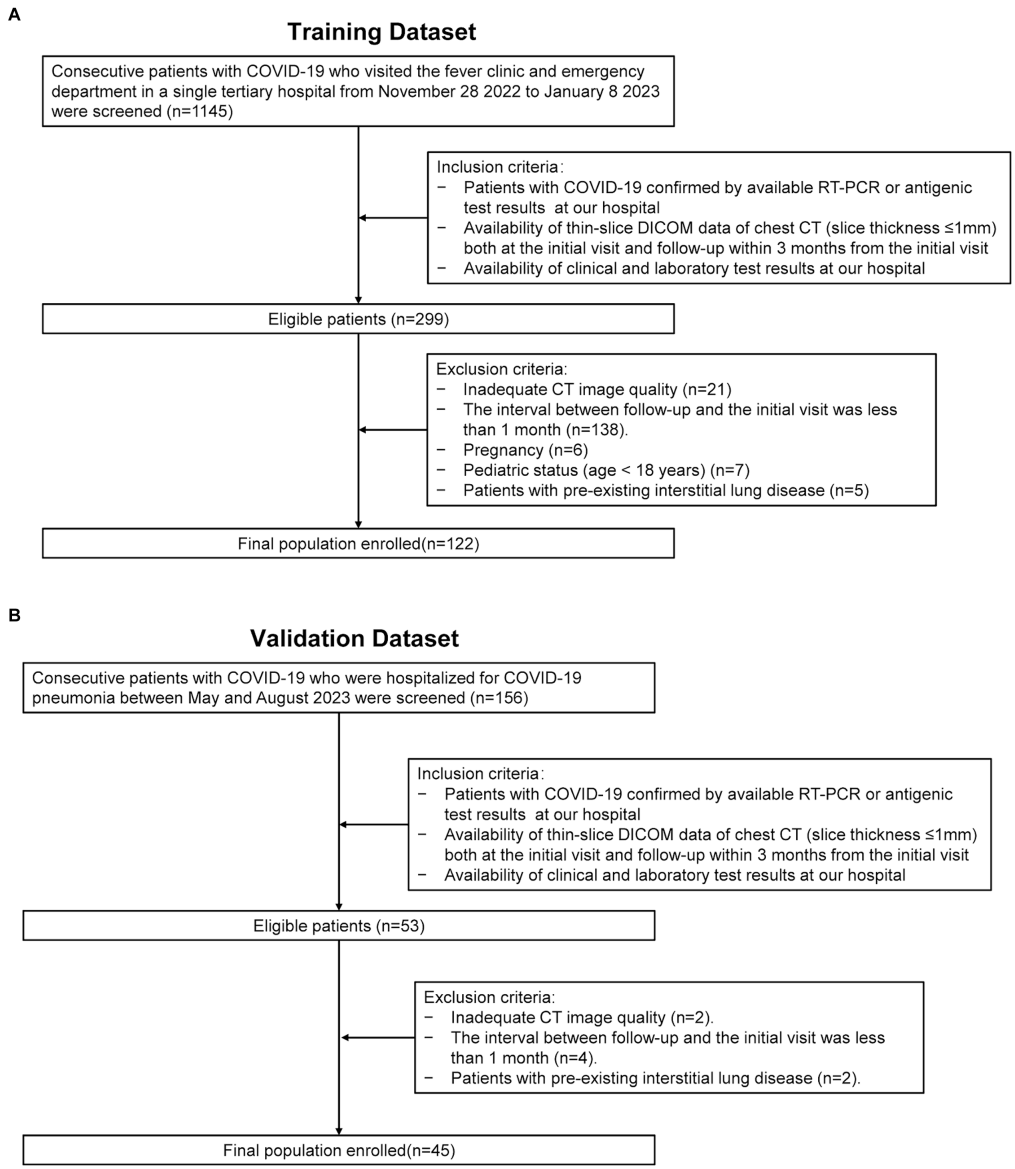
Enrollment flowchart of the study. RT-PCR: real-time reverse-transcription polymerase chain reaction. DICOM: thin-slice digital imaging and communications in medicine. **(A)** Depicts the data collection process utilized for the training dataset. **(B)** Illustrates the data collection process for the validation dataset.

This retrospective study reviewed consecutive patients from two time periods for model development and validation purpose respectively: (1) patients who visited the fever clinic and emergency department at our hospital during the Omicron outbreak from November 28, 2022, to January 8, 2023 (Figure 1A); and (2) patients who were hospitalized for COVID-19 pneumonia between May and August 2023 (Figure 1B). The inclusion criteria were as follows: (1) patients with COVID-19 confirmed by real-time positive polymerase chain reaction (RT-PCR) or antigenic test results at our hospital; (2) the availability of thin-slice digital imaging and communications in medicine (DICOM) data of the chest CT (slice thickness ≤ 1 mm) both at the initial visit and follow-up within 3 months of the initial visit; and (3) the availability of clinical and laboratory test results at our hospital. The exclusion criteria were as follows: (1) patients with an inadequate CT image quality for a quantitative analysis; (2) patients whose interval between follow-up and the initial visit was less than 1 month; (3) pregnant patients; (4) pediatric patients (age < 18 years) and; and (5) patients with pre-existing interstitial lung diseases.

### 2.2 Data collection and CT image acquisition

The information was collected using a digital hospital information system (HIS) that included the demographic data (age and gender), preexisting comorbidities, the initial symptoms, the initial laboratory findings, and the clinical type at baseline. The initial clinical type was divided into mild/moderate and severe/critical classifications by a senior physician who specialized in respiratory diseases. Additional information about data collection can be found in Supplementary materials.

The most severe chest CT images obtained within a 15-day period from the first hospital visit were selected as the initial images. Follow-up chest CT images were then acquired 1–3 months after the initial scan to evaluate disease progression or improvement. The chest CT scan was conducted in a single breath-hold using several multi-detector CT (MDCT) scanners: Somatom Definition Flash or Somatom Force (Siemens, Forchheim, Germany), Discovery CT750 HD (General Electric, Milwaukee, WI) or IQon CT (Philips, The Netherlands). The patients were all scanned in the supine position, with the tube voltage set to 120 kV, and with the automatic tube current exposure-control technology. All images were reconstructed in the axial plane. Images were transmitted to the Picture Archiving and Communication System (PACS) (GE Healthcare, Chicago, IL, United States) for further data analysis.

### 2.3 Qualitative and semi-quantitative image evaluation

**Figure 2:**
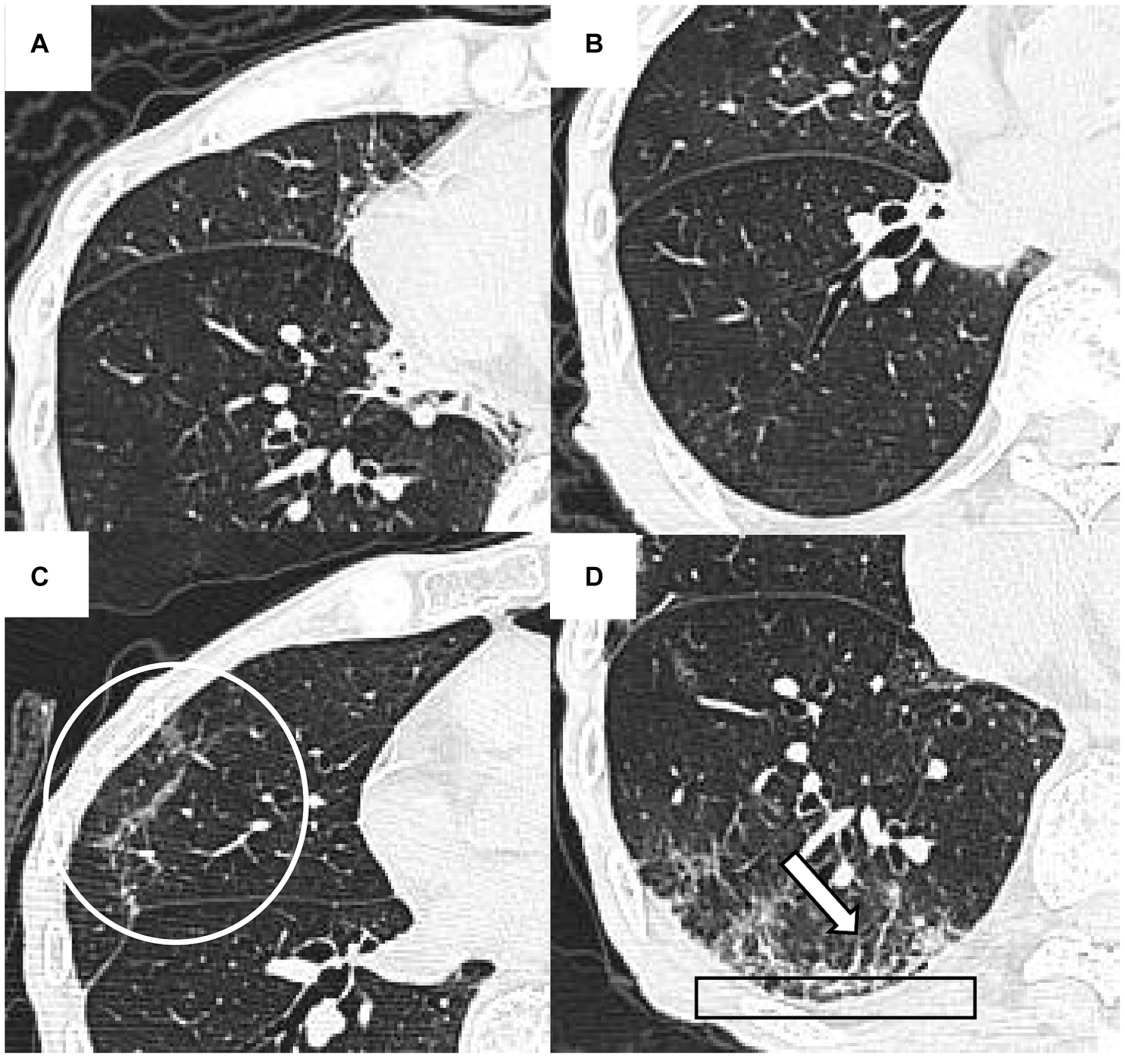
The initial and 48-day follow-up CT findings of a 93-year-old man with COVID-19 pneumonia. The initial CT scans **(A,B)** did not reveal any signs of fibrotic interstitial lung abnormalities (ILAs). However, the follow-up CT **(C,D)** showed fibrotic ILAs at the identical anatomical level. These changes include architectural distortion (indicated by a circle in **C**), reticulation (outlined by a rectangle in **D**), and traction bronchiectasis and/or bronchiolectasis (marked by an arrow in **D**).

The image analysis was performed using the preselected lung (width, 1,200 HU; level, −600 HU) and mediastinal (width, 450 HU; level, 50 HU) window settings. Two senior radiologists (19 years, 30 years of experience in thoracic radiology, respectively) independently reviewed the PACS system images, blind to patients’ clinical or laboratory data. Ambiguous findings were resolved by discussion and consensus. CT findings were defined by the Fleischner Society glossary (21). Signs of fibrotic ILAs were recorded in the presence of reticulation, traction bronchiectasis and/or bronchiolectasis, architectural distortion, or honeycombing (Figure 2) (22, 23). Then, patients were classified into fibrotic group and nonfibrotic group based on whether the fibrotic ILAs were present in the follow-up CT images.

A semi-quantitative CT score was used to quantify the initial pulmonary involvement by two senior radiologist specialized in chest imaging (24). Involvement in each of the five lung lobes was rated on a scale: 0 (no disease), 1 (<5% involvement), 2 (5–24%), 3 (25–49%), 4 (50–74%), and 5 (>75%). The total CT score, summing individual lobe scores, ranged from 0 (no disease) to 25 (maximum involvement). To validate the reliability of the CT scores, the Intraclass Correlation Coefficient (ICC) was calculated to measure the consistency between the two radiologists’ assessments.

### 2.4 Quantitative chest CT analysis

**Figure 3:**
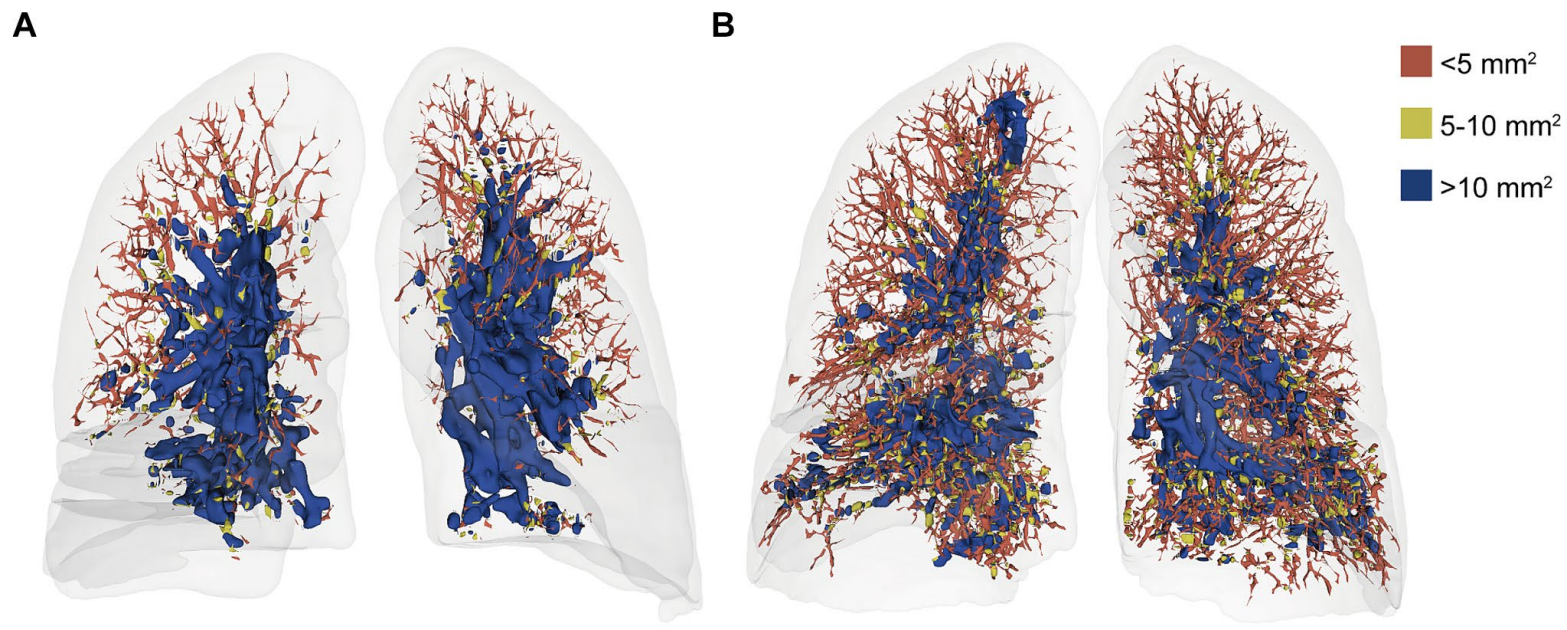
Volume-rendered chest CT images of a 52-year-old woman at initial **(A)** and follow-up **(B)** with color-coded segmentation of the pulmonary vascular cross-sectional area. a. the initial CT with a BV5% of 27.76%. b. the follow-up CT with a BV5% of 51.14%. The color coding denotes the blood volume in vessels with cross-sectional areas <5 mm^2^ (BV5), between 5 and 10 mm^2^ (BV5–10), and > 10 mm^2^ (BV10). In addition, the BV5% is the percentage of the volume of blood contained in vessels with a cross-sectional area less than 5 mm^2^.

A neural network model, based on the U-Net architecture, was employed for the automatic segmentation of pulmonary blood vessels (25). The detailed methodology of this model is elaborated in Supplementary materials. Subsequently, the segmented blood vessels were categorized into three groups based on their cross-sectional areas: BV5 for the blood volume in vessels with a cross-sectional area less than 5 mm^2^, BV5–10 for those with a cross-sectional area between 5 and 10 mm^2^, and BV10 for vessels with a cross-sectional area larger than 10 mm^2^ (Figure 3). The percentages of blood volume within these categories, BV5%, BV5–10%, and BV10%, were computed relative to the total blood volume in the lung.

**Figure 4:**
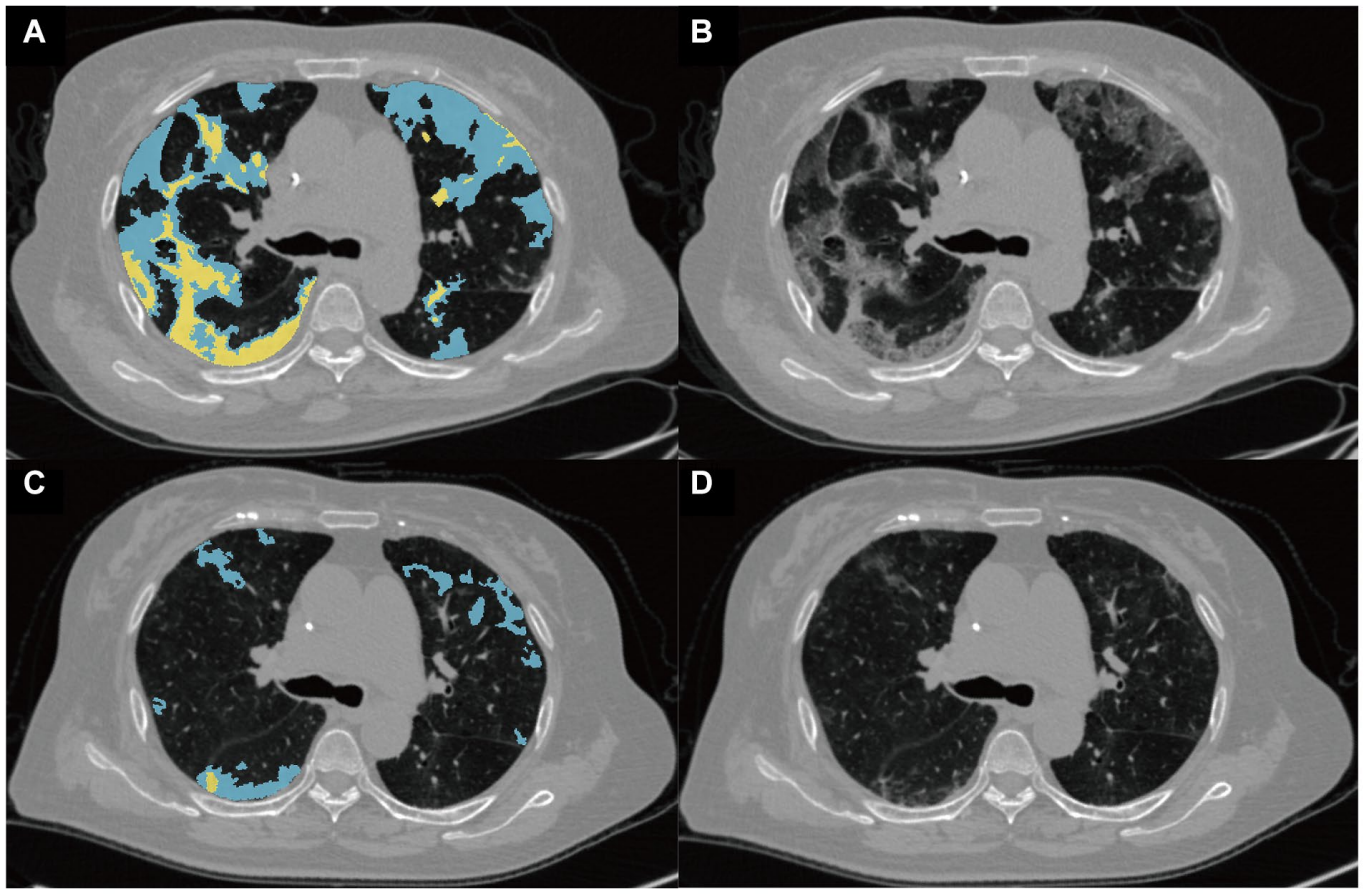
Representative images from the quantitative analysis of pneumonia lesions. Pneumonia lesions at the baseline **(A)** and follow-up **(B)** CT scans of a 75-year-old woman with critical COVID-19 were segmented into ground-glass opacity (GGO) and consolidation, shown in blue and yellow, respectively. The raw baseline and follow-up images of this patient are shown in **(C,D)**, respectively.

The detection, segmentation, and quantification of pneumonia lesions, including consolidation and ground-glass opacity (GGO), were automated using an AI system combining MVP-Net and 3D U-Net (Figure 4). The methodology is detailed in Supplementary materials. The quantitative parameters included the pneumonia volume (PV in ml), the mean attenuation of pneumonia lesions (PA in HU), the GGO volume (GV in ml), and the consolidation volume (CV in ml) in both lungs. In addition, the pneumonia mass (PM in g) was calculated as follows: PM (g) = PV (ml) * [PA(HU) + 1000]/1,000.

### 2.5 Statistical analysis

Statistical analyses were executed in R (version 4.3.1), with categorical variables analyzed via the Chi-square test, normally distributed continuous data by t-test, and non-normally distributed data by the Wilcoxon rank-sum test. Adjustments for multiple tests used the false discovery rate (FDR) method, and missing laboratory data were handled through multiple imputation with R’s “mice” package.

A receiver operating characteristic (ROC) curve analysis was performed for all the above quantitative CT parameters and the CT score to compare their effectiveness in predicting fibrotic ILAs. And the optimal cutoff values were identified to maximize the Youden index. The area under the curve (AUC), accuracy, precision, specificity, sensitivity, and F1 score were computed.

Univariate logistic regression examined the association of these CT, demographic, and laboratory parameters with fibrotic ILAs, removing unevenly distributed variables including comorbidity along with neutrophil and lymphocyte counts. Following this, a multivariate logistic regression model incorporated age, gender, clinical type, anemia, and raised hsCRP levels as covariates to determine if the CT parameters could independently predict fibrotic ILAs. The Delong test compared AUC values among CT parameters.

## 3 Results

### 3.1 Demographic, clinical and laboratory characteristics of the training dataset

As shown in Table 1, a total of 122 patients [73 men, 49 women; age median 68.50 (60.00, 78.75) years] were enrolled in this study. No patients reported previous infection with SARS-CoV-2.

**Table 1:** Group comparison of demographic and clinical characteristics.

Characteristics	All cohort	Nonfibrotic cohort	Fibrotic cohort	*p*
Number of cases	122	54	68	/
Sex				0.07
Male	73 (59.84)	27 (50.00)	46 (67.65)	/
Female	49 (40.16)	27 (50.00)	22 (32.35)	/
Age [median (IQR), years]	68.50 [60.00, 78.75]	64.00 [51.75, 69.00]	74.50 [66.00, 84.25]	<0.001*
Clinical type				<0.001*
Mild/moderate	73 (59.84)	44 (81.48)	29 (42.65)	/
Severe/critical	49 (40.16)	10 (18.52)	39 (57.35)	/
Comorbidity	115 (94.26)	47 (87.04)	68 (100.00)	0.008*
Diabetes	33 (27.05)	12 (22.22)	21 (30.88)	0.39
Cardiovascular disease	60 (49.18)	23 (42.59)	37 (54.41)	0.27
Cerebral artery disease	18 (14.75)	5 (9.26)	13 (19.12)	0.20
Chronic lung disease	40 (32.79)	13 (24.07)	27 (39.71)	0.10
Chronic kidney disease	14 (11.48)	4 (7.41)	10 (14.71)	0.33
Chronic liver disease	2 (1.64)	0 (0.00)	2 (2.94)	0.58
Immunocompromised status or malignancies	58 (47.54)	23 (42.59)	35 (51.47)	0.43

Except where indicated, the data are the numbers of patients, with percentages in parentheses. IQR: Interquartile range. *Denotes *p* < 0.05.

The median and IQR for the time interval between the follow-up CT and initial visit CT was 54 days [43, 75], respectively. Based on whether *de Novo* fibrotic ILAs were present on the follow-up CT (Supplementary Table S1), all patients were divided into a nonfibrotic group (*n* = 54) and a fibrotic group (*n* = 68).

In the fibrotic group, patients were generally older [median (IQR): 74.50 (66.00, 84.25) vs. 64.00 (51.75, 69.00), *p <* 0.001], more frequently categorized as severe/critical at their initial visit (57.35% vs. 18.52%, *p <* 0.001), and more likely to possess at least one pre-existing comorbidity (100% vs. 87.04%, *p =* 0.008) than those in the nonfibrotic group (Table 1).

A comparison of the clinical and laboratory test results and initial clinical symptoms is presented in Supplementary Tables S2, S3, respectively. Anemia (47.06% vs. 14.81%, *p =* 0.001) and increased hsCRP (51.47% vs. 24.07%, *p =* 0.01) were more prevalent in the fibrotic group compared to the nonfibrotic group (Supplementary Table S2). Supplementary Table S3 shows that the fibrotic group exhibited more observations of chest tightness (50.00% vs. 27.78%, *p =* 0.02) than the nonfibrotic group.

### 3.2 Comparison of the qualitative CT features, the CT score, and quantitative CT parameters at baseline within the training dataset

The ICC for the CT score between the two radiologists was 0.99, indicating nearly perfect agreement. The Bland–Altman plot (Supplementary Figure S1) also demonstrated excellent interobserver reliability in the quantitative assessment of lung involvement using the CT score.

As shown in Table 2, patients in the fibrotic group had much higher CT score than those in the nonfibrotic group [median (IQR): 14.00 (7.75, 17.00) vs. 7.00 (3.00, 9.75), *p <* 0.001]. The patients in the fibrotic group were more likely to have higher prevalences of GGO (100.00% vs. 83.33%, *p =* 0.002) and consolidation (52.94% vs. 27.78%, *p =* 0.009) at the initial CT than those in the nonfibrotic group.

**Table 2:** Group comparison of initial CT features.

CT features	All cohort	Nonfibrotic cohort	Fibrotic cohort	*p*
Number of cases	122	54	68	/
GGO	113 (92.62)	45 (83.33)	68 (100.00)	0.002*
consolidation	51 (41.80)	15 (27.78)	36 (52.94)	0.009*
CT score				
LUL (median [IQR])	2.00 [1.00, 3.00]	1.00 [1.00, 2.00]	2.00 [1.00, 3.00]	<0.001*
LLL [median (IQR)]	2.00 [2.00, 3.00]	2.00 [1.00, 3.00]	3.00 [2.00, 4.00]	<0.001*
RUL [median (IQR)]	2.00 [1.00, 3.00]	1.00 [0.00, 2.00]	2.00 [2.00, 3.00]	<0.001*
RML [median (IQR)]	2.00 [0.00, 3.00]	1.00 [0.00, 2.00]	2.50 [1.75, 3.00]	<0.001*
RLL [median (IQR)]	2.00 [2.00, 4.00]	2.00 [1.00, 2.00]	3.00 [2.00, 4.00]	<0.001*
Total [median (IQR)]	9.50 [6.25, 15.00]	7.00 [3.00, 9.75]	14.00 [7.75, 17.00]	<0.001*
Lober number [median (IQR)]	5.00 [4.00, 5.00]	4.50 [2.00, 5.00]	5.00 [4.00, 5.00]	0.005*
Quantitative CT parameters				
PV [median (IQR)]/ml	182.10 [44.25, 578.07]	72.81 [14.12, 194.55]	337.11 [113.45, 858.41]	<0.001*
CV [median (IQR)]/ml	49.08 [12.87, 146.80]	18.36 [2.67, 48.78]	99.53 [38.23, 293.41]	<0.001*
GV [median (IQR)]/ml	133.53 [28.47, 344.33]	52.41 [10.41, 134.38]	225.96 [73.63, 500.65]	<0.001*
PM [median (IQR)]/g	100.75 [25.22, 330.27]	42.29 [7.90, 103.04]	193.15 [67.14, 527.08]	<0.001*
BV5% [mean (SD)]	39.90% (11.07%)	43.25% (10.65%)	37.24% (10.74%)	0.003*
PA [median (IQR)]/HU	−397.23 [−439.79, −355.83]	−423.30 [−461.94, −358.31]	−385.00 [−432.68, −355.96]	0.11

Except where indicated, the data are the numbers of patients, with percentages in parentheses. GGO, ground-glass opacity; LUL, left upper lobe; LLL, left lower lobe; RUL, right upper lobe; RML, right middle lobe; RLL, right lower lobe; PV, pneumonia volume; CV, consolidation volume; GV, ground-glass opacity volume; PM, pneumonia mass; BV5%, the percentage of the volume of blood contained in vessels with a cross-sectional area lower than 5 mm^**2**^; PA, mean attenuation of pneumonia; IQR, Interquartile range. *Denotes *p* < 0.05.

As for the quantitative CT parameters, compared with the nonfibrotic group, the patients in the fibrotic group had higher levels of baseline PV, CV, GV, and PM (all *p <* 0.001). For the blood volume distribution, patients in the fibrotic group had significantly lower BV5% compared with patients in the nonfibrotic group (*p =* 0.003) on the baseline CT during the acute infection phase.

### 3.3 Comparison of the CT features between the initial and follow-up chest CT in the training dataset

At the 3-month follow-up chest CT (Supplementary Table S1), persistent GGO was observed in 103 of 122 patients (84.43%), while 15 of 122 patients (12.30%) exhibited consolidation. Fibrotic ILAs were present in 68 of 122 patients (55.74%).

As detailed in Table 3, compared with the initial chest CT, the percentage of patients with consolidation (41.80% vs.12.30%, *p <* 0.001) was significantly reduced in the follow-up CT. Furthermore, the follow-up CT showed notable Decreases in the CT score, PV, CV, GV, PM and PA (all *p <* 0.001).

**Table 3:** Comparison of CT Findings and scores between the initial and follow-up.

CT features	Baseline CT	Follow-up CT	*p*
Number of cases	122	122	/
GGO (%)	113 (92.62)	103 (84.43)	0.07
Consolidation (%)	51 (41.80)	15 (12.30)	<0.001*
CT score			
LUL (median [IQR])	2.00 [1.00, 3.00]	1.00 [0.00, 2.00]	0.01*
LLL (median [IQR])	2.00 [2.00, 3.00]	2.00 [0.00, 3.00]	0.01*
RUL (median [IQR])	2.00 [1.00, 3.00]	1.00 [0.00, 2.00]	0.01*
RML (median [IQR])	2.00 [0.00, 3.00]	1.00 [0.00, 2.00]	0.002*
RLL (median [IQR])	2.00 [2.00, 4.00]	2.00 [1.00, 3.00]	0.007*
Total (median [IQR])	9.50 [6.25, 15.00]	6.00 [2.00, 13.00]	0.002*
Lober number(median [IQR])	5.00 [4.00, 5.00]	4.00 [2.00, 5.00]	0.002*
AI derived CT features			
PV (median [IQR])/ml	182.10 [44.25, 578.07]	49.41 [4.49, 173.18]	<0.001*
CV (median [IQR])/ml	49.08 [12.87, 146.80]	9.63 [1.05, 37.51]	<0.001*
GV (median [IQR])/ml	133.53 [28.47, 344.33]	37.16 [3.84, 136.85]	<0.001*
PM (median [IQR])/g	100.75 [25.22, 330.27]	25.22 [2.38, 93.45]	<0.001*
BV5% (mean [SD])	40.94% [31.86, 48.77%]	47.12% [35.91, 54.42%]	<0.001*
PA (median [IQR])/HU	133.53 [28.47, 344.33]	37.16 [3.84, 136.85]	<0.001*

Except where indicated, the data are the numbers of patients, with percentages in parentheses. IQR, Interquartile range; GGO, ground-glass opacity; LUL, left upper lobe; LLL, left lower lobe; RUL, right upper lobe; RML, right middle lobe; RLL, right lower lobe; PV, pneumonia volume; CV, consolidation volume; GV, ground-glass opacity volume; PM: pneumonia mass; BV5%: the percentage of the volume of blood contained in vessels with cross-sectional areas less than 5 mm^2^; PA, the attenuation of pneumonia. *Denotes *p* < 0.05.

### 3.4 Follow-up findings of the microcirculation in the training dataset

**Figure 5:**
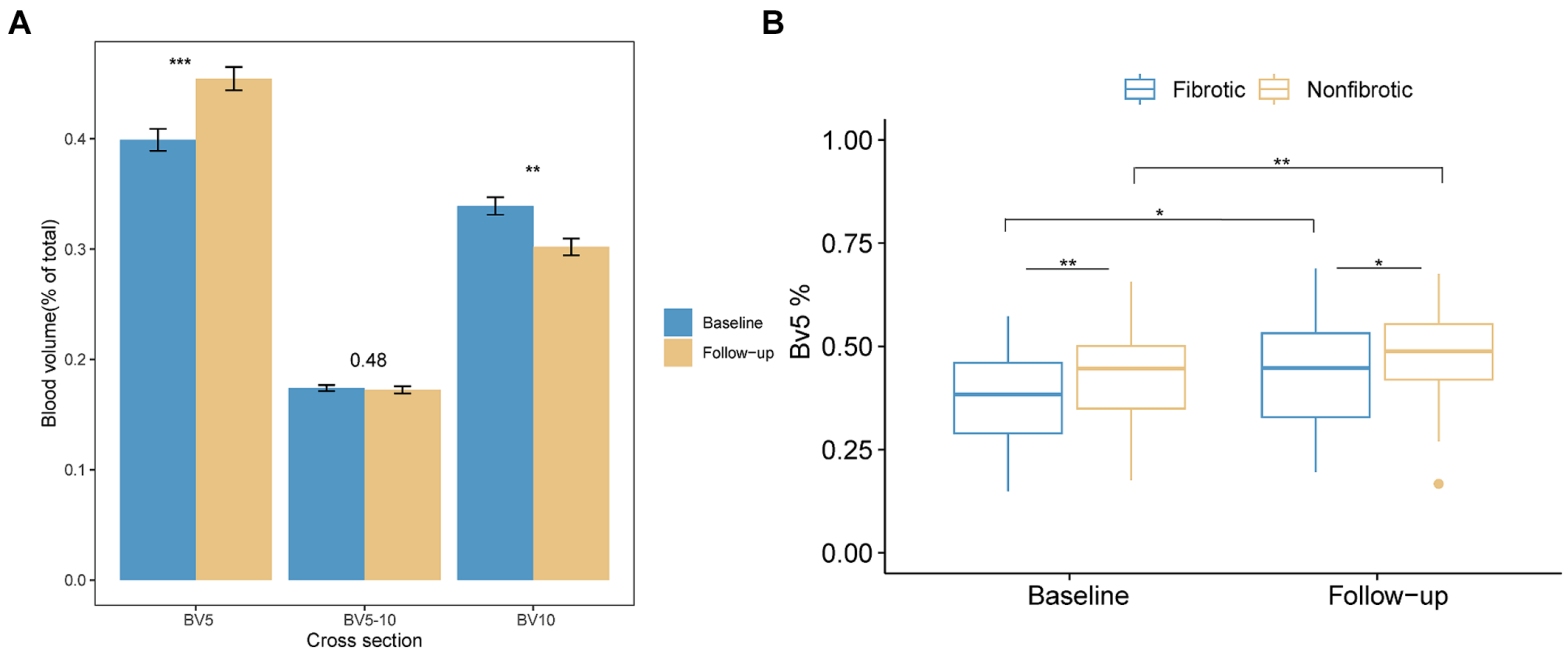
Alterations in the distribution of the pulmonary vascular volume are presented as follows. **(A)** The bar plot illustrates variations in the proportion of blood volume across the three distinct groups at the initial and follow-up CT scans. The three groups included BV5 for blood volume in vessels with cross-sectional areas less than 5 mm^2^, BV5–10 for those with cross-sectional areas between 5 and 10 mm^2^, and BV10 for those with cross-sectional areas larger than 10 mm^2^. **(B)** The boxplot depicts the percentage of blood volume in vessels with cross-sectional areas less than 5 mm^2^ relative to the total pulmonary blood volume (BV5%) in the non-fibrotic and fibrotic groups in the initial and follow-up CT. *Denotes *p <* 0.05; **denotes *p <* 0.01; ***denotes *p <* 0.001.

In contrast to PV, BV5% demonstrated a significant increase in the follow-up CT (*p <* 0.001), while BV10% had a notable Decline in the follow-up CT compared with the initial CT (*p <* 0.01) (Figure 5). Concurrently, the BV5% of the patients in the fibrotic group was significantly lower at both the initial (*p =* 0.008) and follow-up CT (*p =* 0.048) compared to that of the nonfibrotic group (Figure 5).

### 3.5 Predictive value of quantitative CT parameters versus CT score for fibrotic ILAs

The findings from the ROC curve analysis and the optimal cutoff values for all CT parameters are shown in Supplementary Table S4.

The univariate logistic regression analysis (Supplementary Table S5) showed that age over 68.5 years (*p <* 0.001), being male (*p =* 0.0497), severe/critical clinical type (*p <* 0.001), anemia (*p <* 0.001), and increased hsCRP (*p =* 0.02) at the initial visit were associated with a greater likelihood of fibrotic ILAs.

Using these variables as covariates, a multivariate logistic regression analysis was performed for each CT parameter. Table 4 shows the multivariate outcomes that indicated that PV (adjusted OR 3.28, 95% CI: 1.20–9.31, *p =* 0.02), CV (adjusted OR 3.77, 95% CI: 1.37–10.75, *p =* 0.01), GV (adjusted OR 3.38, 95% CI: 1.26–9.38, *p =* 0.02), the CT score (adjusted OR 12.06, 95% CI: 3.15–58.89, *p <* 0.001), and PM (adjusted OR 3.58, 95% CI: 1.28–10.46, *p =* 0.02) were independent predictors for fibrotic ILAs at their respective cutoff values. Conversely, BV5% at its cutoff value, despite showing a negative association with fibrotic ILAs in the initial univariate logistic regression analysis (OR 0.33, 95% CI: 0.16–0.70, *p =* 0.004), was not an independent predictor in the subsequent multivariate models after adjustment for the above confounders (*p =* 0.50).

**Table 4:** Univariate and multivariate logistic regression analysis of CT quantitative parameters and CT score.

CT features	Unadjusted model (*n* = 122)			Adjusted model (*n* = 122)		
	Odd ratio	95%CI	*p*	Odd ratio	95%CI	*p*
PV > cutoff	6.39	2.88–14.19	<0.001*	3.28	1.20–9.31	0.02*
CV > cutoff	7.12	3.2–15.88	<0.001*	3.77	1.37–10.75	0.01*
GV > cutoff	6.59	2.95–14.74	<0.001*	3.38	1.26–9.38	0.02*
CT score > cutoff	14.92	4.85–45.94	<0.001*	12.06	3.15–58.89	<0.001*
PM > cutoff	7.06	3.14–15.85	<0.001*	3.58	1.28–10.46	0.02*
BV5% > cutoff	0.33	0.16–0.70	0.004*	0.72	0.29–1.87	0.50
PA > cutoff	2.58	1.22–5.47	0.01*	1.88	0.75–4.77	0.18

Adjusted models used age > 68.50, sex, clinical type, and abnormal laboratory tests (anemia and elevated high-sensitivity C-reactive protein level (hsCRP)) as covariates. The median of age, 68.50 years, was used as the age cutoff value. PV, pneumonia volume; CV, consolidation volume; GV, ground-glass opacity volume; PM, pneumonia mass; BV5%, the percentage of the volume of blood contained in vessels with cross-sectional areas less than 5 mm^2^; PA, the attenuation of pneumonia. *Denotes *p* < 0.05.

**Figure 6:**
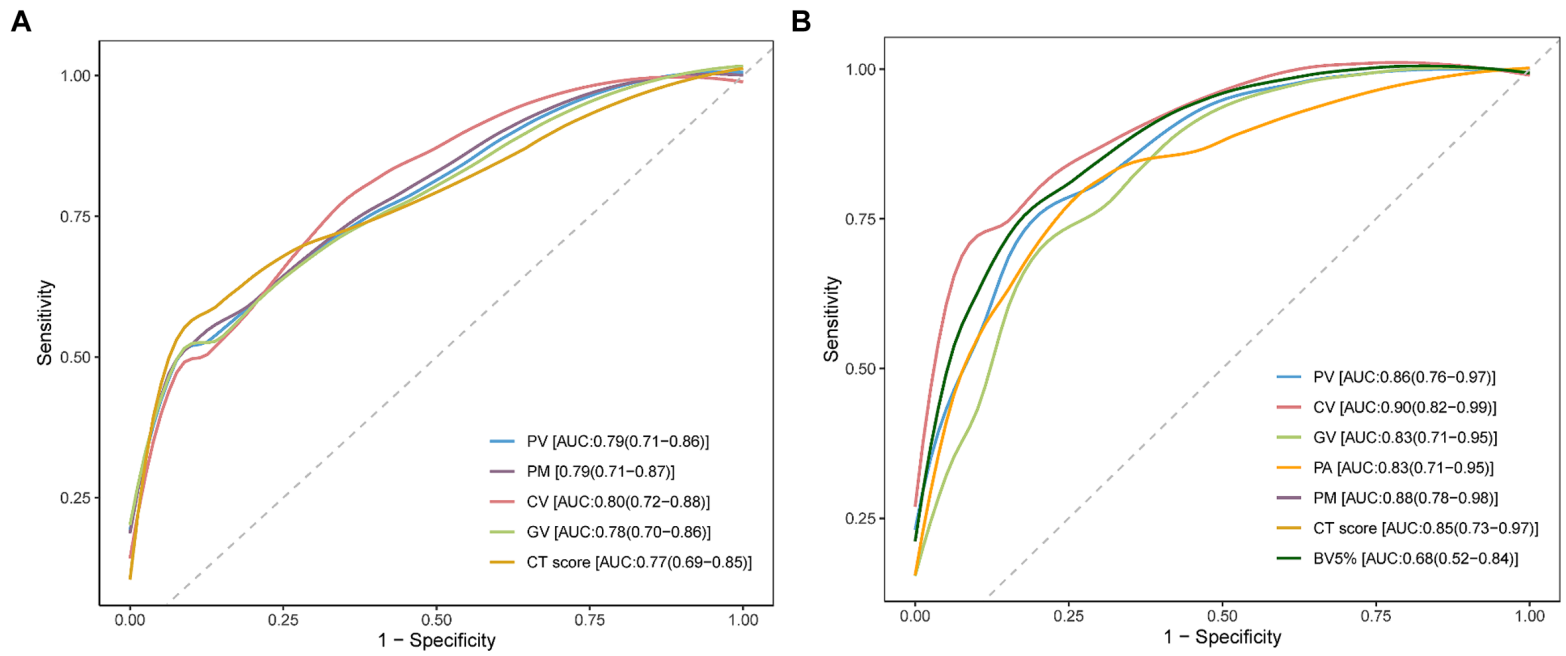
Receiver operating characteristic (ROC) curve analysis for CT parameters. **(A)** ROC curve analysis was performed individually for pneumonia volume (PV), pneumonia mass (PM), consolidation volume (CV), ground-glass opacity volume (GV), and the CT score to assess their effectiveness in predicting the development of post-COVID-19 fibrotic interstitial lung abnormalities (ILAs). The area under the curve (AUC) values are noted in the bottom right corner of the image. **(B)** This panel shows the ROC curve analysis for PV, PM, CV, GV, mean attenuation of pneumonia lesions (PA), the percentage of blood volume in vessels with a cross-sectional area less than 5 mm^2^ (BV5%), and the CT score, conducted on the validation dataset to further validate the predictive capabilities of these CT parameters. The AUC values for these parameters were annotated in the bottom right corner of the image.

The ROC curves of the PV, PM, CV, GV, and the CT score are shown in Figure 6A. As detailed in Supplementary Table S4, CV demonstrated the highest AUC value (0.80, 95% CI: 0.72–0.88) compared with PV (0.79, 95% CI: 0.71–0.86), GV (0.78, 95% CI: 0.70–0.86), PM (0.79, 95% CI: 0.71–0.87), and the CT score (0.77, 95% CI: 0.69–0.85). However, there was no significant difference among the AUC values of these AI-derived CT parameters and the CT score examined by the Delong test (all *p* > 0.05). In addition, the CT score had the lowest sensitivity (0.54) relative to these AI-derived quantitative CT parameters (PV: 0.69, CV: 0.75, GV: 0.68, PM: 0.69).

### 3.6 Validation of the predictive role of quantitative CT parameters for fibrotic ILAs

As detailed in Supplementary Table S6, the validation dataset revealed that 26 out of 45 patients developed *de Novo* fibrotic ILAs in follow-up CT scans.

The ROC curve analysis and optimal cutoff values for all CT parameters are presented in Supplementary Table S7. Univariate logistic regression analysis (Supplementary Table S8) identified that the severe/critical clinical type (*p* = 0.03), elevated WBC (*p* = 0.01), and increased neutrophil count (*p* = 0.01) at the initial visit were significantly associated with a higher likelihood of developing fibrotic ILAs.

Building on these findings, multivariate logistic regression analyses were conducted using these variables as covariates for each CT parameter. The results, shown in Supplementary Table S9, indicate that PV, CV, GV, PM, PA, BV5%, and the CT score are all independent predictors of fibrotic ILAs at their respective cutoff values (all adjusted *p* < 0.05).

The ROC curves depicted in Figure 6B demonstrate the AUC values for various CT parameters: PV (0.86, 95% CI: 0.76–0.97), CV (0.90, 95% CI: 0.82–0.99), GV (0.83, 95% CI: 0.71–0.95), PM (0.88, 95% CI: 0.78–0.98), PA (0.83, 95% CI: 0.71–0.95), BV5% (0.68, 95% CI: 0.52–0.84), and the CT score (0.85, 95% CI: 0.73–0.97). Despite the differences in AUC values, the DeLong test indicated no significant statistical differences among these AI-derived CT parameters and the CT score (all *p* > 0.05).

## 4 Discussion

This retrospective study revealed that 55.74% patients exhibited fibrotic ILAs in the 3-month follow-up. Patients with fibrotic ILAs tended to be older, were more likely initially classified as severe/critical, and had a higher incidence of pre-existing comorbidities, compared to those without fibrotic ILAs. Moreover, anemia and elevated levels of hsCRP at the initial visit were more commonly observed in the fibrotic ILAs group. Independent predictors of fibrotic ILAs included PV, CV, GV, and PM as well as the initial CT score, with validation confirmed in the subsequent dataset. Based on the ROC analysis, the AUC values among these five parameters did not show significant differences. In addition, there was a notable redistribution of the pulmonary blood volume in the follow-up CT, as evidenced by an increased BV5% and a Decreased BV10%. Additionally, patients with fibrotic ILAs were observed to have a consistently lower BV5% at both the initial and follow-up CT compared to those who did not develop fibrotic ILAs.

The proportion of patients with fibrotic changes post COVID-19 in our study was higher than that in some studies (ranging from 1.6 to 28.4%) (22, 23, 26–28). This discrepancy could be attributed to varying definitions of fibrotic changes on CT. Some studies have limited the definition of fibrosis to the occurrence of volume loss and traction bronchiectasis and/or bronchiolectasis (27), which led to a lower incidence of fibrotic ILAs. Also, these different results May have been related to the younger age of their population, compared with our older population of patients who had a higher rate of pre-existing comorbidities (23). In addition, most patients in our study had no prior exposure to the virus, making them more severe at baseline.

Previous studies have demonstrated that the CT score correlates with inflammation Markers and outcomes, serving as an indicator of lung inflammation severity (29). While the precise mechanism of fibrotic ILAs post-COVID-19 remains unclear, it is thought to be driven by persistent, severe inflammation and the release of fibrosis-promoting factors such as TGF-*β* (30). This suggests that the baseline CT score May also be a predictive Marker for post-COVID fibrosis, which was confirmed by recent researches (13, 31). In our study, the PV, CV, GV, PM, and the CT score were all independent predictors of fibrotic ILAs. Based on the ROC curve analysis, the AI-derived quantitative CT parameters matched the predictive capacity of the CT score for fibrotic ILAs. Furthermore, the sensitivities of these AI-derived parameters were all higher than the CT score, suggesting that the AI-derived parameters May be more sensitive predictors relative to the CT score. In addition, AI tools can offer the potential for faster, more stable, and more objective assessments. The predictive performance of the proposed AI-derived quantitative CT parameters was further validated in COVID-19 patients from a later time period in a broader clinical setting to demonstrate its generalizability within a single center.

In our study, the AI-based pulmonary segmentation tool was utilized for the first time to longitudinally evaluate changes in the pulmonary blood volume distribution after COVID-19. A significant increase in BV5% and a Decrease in BV10% were observed in the follow-up CT, suggesting a post-infection recovery of microcirculation dysfunction. However, recently, Mohamed I et al. reported that 87.4% patients exhibited perfusion abnormalities in the 6-month follow-up dual-energy CT (DECT) angiography, which suggested that lung microcirculation dysfunction can persist up to 6 months following COVID-19 infection (32). Also, Kuchler et al. found that persistent endothelial dysfunction was related to ongoing symptoms in patients after COVID-19 infection (33). Correspondingly, in our study, the BV5% in patients with fibrotic ILAs was lower than that in patients without fibrotic ILAs at the follow-up CT, suggesting persistent microcirculation dysfunction in patients with fibrotic ILAs. However, the relationship between persistent microcirculation dysfunction and fibrotic ILAs has not yet been investigated.

Our study had several limitations. First, the study was performed in a single-center setting with a small number of study participants. Second, a correlative analysis of the temporal changes on the CT scans with symptoms and lung function was not performed. Third, a longer period of monitoring is required to estimate whether the fibrotic changes and microcirculation dysfunction were irreversible.

In conclusion, our study demonstrated that quantitative CT parameters, including PV, CV, GV, and PM, were independent predictors for fibrotic ILAs with predictive capabilities comparable to the CT score. In addition, the vascular segmentation results showed ongoing microcirculation dysfunction in patients with fibrotic ILAs, which suggests a potential correlation between BV5% and fibrotic ILAs. Thus, our results indicate that an AI-based CT quantitative analysis might be an effective and important tool in the management of post-COVID-19 sequel.

## Data Availability

The raw data supporting the conclusions of this article will be made available by the authors, without undue reservation.

## References

[1] Atabati E Dehghani-Samani A Mortazavimoghaddam SG. Association of COVID-19 and other viral infections with interstitial lung diseases, pulmonary fibrosis, and pulmonary hypertension: a narrative review. Can J Respir Ther. (2020) 56:70–8. doi: 10.29390/cjrt-2020-021 PMC7690312 33274259

[2] Huang WJ Tang XX. Virus infection induced pulmonary fibrosis. J Transl Med. (2021) 19:496. doi: 10.1186/s12967-021-03159-9 34876129 PMC8649310

[3] Sheng G Chen P Wei Y Yue H Chu J Zhao J. Viral infection increases the risk of idiopathic pulmonary fibrosis: a Meta-analysis. Chest. (2020) 157:1175–87. doi: 10.1016/j.chest.2019.10.032, PMID: 31730835 PMC7214095

[4] Hata A Schiebler ML Lynch DA Hatabu H. Interstitial lung abnormalities: state of the art. Radiology. (2021) 301:19–34. doi: 10.1148/radiol.2021204367, PMID: 34374589 PMC8487219

[5] Hatabu H Hunninghake GM Richeldi L Brown KK Wells AU Remy-Jardin M. Interstitial lung abnormalities detected incidentally on CT: a position paper from the Fleischner society. Lancet Respir Med. (2020) 8:726–37. doi: 10.1016/S2213-2600(20)30168-5, PMID: 32649920 PMC7970441

[6] Han X Chen L Fan Y Alwalid O Jia X Zheng Y. Longitudinal assessment of chest CT findings and pulmonary function after COVID-19 infection. Radiology. (2023) 307:e222888. doi: 10.1148/radiol.222888, PMID: 36786698 PMC9969419

[7] Dong E Du H Gardner L. An interactive web-based dashboard to track COVID-19 in real time. Lancet Infect Dis. (2020) 20:533–4. doi: 10.1016/S1473-3099(20)30120-1, PMID: 32087114 PMC7159018

[8] Al-Kuraishy HM Batiha GES Faidah H Al-Gareeb AI Saad HM Simal-Gandara J. Pirfenidone and post-Covid-19 pulmonary fibrosis: invoked again for realistic goals. Inflammopharmacology. (2022) 30:2017–26. doi: 10.1007/s10787-022-01027-6, PMID: 36044102 PMC9430017

[9] Mizera J Genzor S Sova M Stanke L Burget R Jakubec P. The effectiveness of glucocorticoid treatment in post-COVID-19 pulmonary involvement. Pneumonia (Nathan). (2024) 16:2. doi: 10.1186/s41479-023-00123-7, PMID: 38311783 PMC10840187

[10] Lassan S Tesar T Tisonova J Lassanova M. Pharmacological approaches to pulmonary fibrosis following COVID-19. Front Pharmacol. (2023) 14:1143158. doi: 10.3389/fphar.2023.1143158, PMID: 37397477 PMC10308083

[11] Duong-Quy S Vo-Pham-Minh T Tran-Xuan Q Huynh-Anh T Vo-Van T Vu-Tran-Thien Q. Post-COVID-19 pulmonary fibrosis: facts—challenges and futures: a narrative review. Pulm Ther. (2023) 9:295–307. doi: 10.1007/s41030-023-00226-y, PMID: 37209374 PMC10199290

[12] Yasin R Gomaa AAK Ghazy T Hassanein SA Ibrahem RA Khalifa MH. Predicting lung fibrosis in post-COVID-19 patients after discharge with follow-up chest CT findings. Egypt J Radiol Nucl Med. (2021) 52:495. doi: 10.1186/s43055-021-00495-0

[13] Alilou S Zangiabadian M Pouramini A Jaberinezhad M Shobeiri P Ghozy S. Radiological findings as predictors of COVID-19 lung sequelae: a systematic review and Meta-analysis. Acad Radiol. (2023) 30:3076–85. doi: 10.1016/j.acra.2023.06.002, PMID: 37491177 PMC10242153

[14] Yu M Liu Y Xu D Zhang R Lan L Xu H. Prediction of the development of pulmonary fibrosis using serial thin-section CT and clinical features in patients discharged after treatment for COVID-19 pneumonia. Korean J Radiol. (2020) 21:746–55. doi: 10.3348/kjr.2020.0215, PMID: 32410413 PMC7231610

[15] Zhao G Feng Q Chen C Zhou Z Yu Y. Diagnose like a radiologist: hybrid neuro-probabilistic reasoning for attribute-based medical image diagnosis. IEEE Trans Pattern Anal Mach Intell. (2022) 44:7400–16. doi: 10.1109/TPAMI.2021.3130759 34822325

[16] Zhang K Liu X Shen J Li Z Sang Y Wu X. Clinically applicable AI system for accurate diagnosis, quantitative measurements, and prognosis of COVID-19 pneumonia using computed tomography. Cell. (2020) 181:1423–1433.e11. doi: 10.1016/j.cell.2020.04.045, PMID: 32416069 PMC7196900

[17] Zhou HY Yu Y Wang C Zhang S Gao Y Pan J. A transformer-based representation-learning model with unified processing of multimodal input for clinical diagnostics. Nat Biomed Eng. (2023) 7:743–55. doi: 10.1038/s41551-023-01045-x 37308585

[18] Caruso D Guido G Zerunian M Polidori T Lucertini E Pucciarelli F. Postacute sequelae of COVID-19 pneumonia: 6-month chest CT follow-up. Radiology. (2021) 301:E396–405. doi: 10.1148/radiol.2021210834 34313468 PMC8335814

[19] Liu F Zhang Q Huang C Shi C Wang L Shi N. CT quantification of pneumonia lesions in early days predicts progression to severe illness in a cohort of COVID-19 patients. Theranostics. (2020) 10:5613–22. doi: 10.7150/thno.45985, PMID: 32373235 PMC7196293

[20] Morris MF Pershad Y Kang P Ridenour L Lavon B Lanclus M. Altered pulmonary blood volume distribution as a biomarker for predicting outcomes in COVID-19 disease. Eur Respir J. (2021) 58:2004133. doi: 10.1183/13993003.04133-2020, PMID: 33632795 PMC7908189

[21] Hansell DM Bankier AA Mac Mahon H McLoud TC Müller NL Remy J. Fleischner society: glossary of terms for thoracic imaging. Radiology. (2008) 246:697–722. doi: 10.1148/radiol.2462070712 18195376

[22] Watanabe A So M Iwagami M Fukunaga K Takagi H Kabata H. One-year follow-up CT findings in COVID-19 patients: a systematic review and meta-analysis. Respirology. (2022) 27:605–16. doi: 10.1111/resp.14311, PMID: 35694728 PMC9350074

[23] Bocchino M Lieto R Romano F Sica G Bocchini G Muto E. Chest CT-based assessment of 1-year outcomes after moderate COVID-19 pneumonia. Radiology. (2022) 305:479–85. doi: 10.1148/radiol.220019 35536134 PMC9619196

[24] Pan F Ye T Sun P Gui S Liang B Li L. Time course of lung changes at chest CT during recovery from coronavirus disease 2019 (COVID-19). Radiology. (2020) 295:715–21. doi: 10.1148/radiol.2020200370, PMID: 32053470 PMC7233367

[25] Bai Y Wang X Zhou Z Wu Z Feng Q Qi J. *Pulmonary segments segmentation with hierarchical weak labels*. IEEE 20th International Symposium on Biomedical Imaging (ISBI), pp. 1–5. (2023).

[26] Luger AK Sonnweber T Gruber L Schwabl C Cima K Tymoszuk P. Chest CT of lung injury 1 year after COVID-19 pneumonia: the Cov ILD study. Radiology. (2022) 304:462–70. doi: 10.1148/radiol.211670, PMID: 35348379 PMC8988857

[27] Vijayakumar B Tonkin J Devaraj A Philip KEJ Orton CM Desai SR. CT lung abnormalities after COVID-19 at 3 months and 1 year after hospital discharge. Radiology. (2022) 303:444–54. doi: 10.1148/radiol.2021211746, PMID: 34609195 PMC8515207

[28] Bocchino M Rea G Capitelli L Lieto R Bruzzese D. Chest CT lung abnormalities 1 year after COVID-19: a systematic review and Meta-analysis. Radiology. (2023) 308:e230535. doi: 10.1148/radiol.230535, PMID: 37404150

[29] Abd El Megid AGI El Shabrawy M Abdalla AAEHM. Correlation between chest CT severity scoring system with oxygen saturation and laboratory inflammatory markers in adult patients with COVID-19 infection. Egypt J Radiol Nucl Med. (2022) 53:747. doi: 10.1186/s43055-022-00747-7

[30] Giacomelli C Piccarducci R Marchetti L Romei C Martini C. Pulmonary fibrosis from molecular mechanisms to therapeutic interventions: lessons from post-COVID-19 patients. Biochem Pharmacol. (2021) 193:114812. doi: 10.1016/j.bcp.2021.114812, PMID: 34687672 PMC8546906

[31] Lyu P Liu X Zhang R Shi L Gao J. The performance of chest CT in evaluating the clinical severity of COVID-19 pneumonia: identifying critical cases based on CT characteristics. Investig Radiol. (2020) 55:412–21. doi: 10.1097/RLI.0000000000000689 32304402 PMC7173027

[32] Mohamed I de Broucker V Duhamel A Giordano J Ego A Fonne N. Pulmonary circulation abnormalities in post-acute COVID-19 syndrome: dual-energy CT angiographic findings in 79 patients. Eur Radiol. (2023) 33:4700–12. doi: 10.1007/s00330-023-09618-9 37145145 PMC10129318

[33] Kuchler T Günthner R Ribeiro A Hausinger R Streese L Wöhnl A. Persistent endothelial dysfunction in post-COVID-19 syndrome and its associations with symptom severity and chronic inflammation. Angiogenesis. (2023) 26:547–63. doi: 10.1007/s10456-023-09885-6 37507580 PMC10542303

