# Supplementary material for: U-Net-based computed tomography quantification of viral pneumonia can predict fibrotic interstitial lung abnormalities at 3-month follow-up
Source: Front Med (Lausanne). 2024 Sep 30;11:1435337. doi: 10.3389/fmed.2024.1435337 (PMC11471527; doi:10.3389/fmed.2024.1435337)
Supplement: Supplementary file 1 [file Data_Sheet_1.docx]

Supplementary Material

# Supplementary Data

## Data collection and CT image acquisition

The initial laboratory findings were defined as follows: low platelet counts were indicated by numbers under 100×10^9^/L, and anemia was indicated by hemoglobin less than 120 mg/dl for men and 110 mg/dl for women. Elevations of high-sensitivity C-reactive protein level (hsCRP) were marked by levels above 50 mg/L. Higher white blood cell (WBC) counts were greater than 9.5×10^9^/L, and elevated neutrophil counts were above 7.5×10^9^/L. Conversely, lymphocyte counts below 0.8×10^9^/L and eosinophil counts below 0.02×10^9^/L were considered low.

And the classification of clinical types of COVID-19 was according to the guideline for the diagnosis and treatment of COVID-19 (trial version 10) established by the China’s National Health Commission on Jan. 2023 (1): Mild patients exhibit mainly upper respiratory symptoms, such as sore throat, cough, and fever, without any lung opacities on chest imaging. Moderate patients should meet following criteria: 1) persistent fever for more than three days and/or symptoms like dyspnea and cough, with a respiratory rate below 30 beats per minute and blood oxygen saturation above 93% without oxygen support. 2) Chest imaging reveals lung opacities. Severe patients meet any of the following: respiratory distress with a respiratory rate above 30 beats per minute; blood oxygen saturation at rest is 93% or less; arterial blood oxygen partial pressure or oxygen saturation is 300 mm Hg or below; lung opacities increase by more than 50% within 24 to 48 hours, indicating progressive worsening of symptoms. Critical patients experience respiratory failure requiring mechanical ventilation, shock, or failure of other organs necessitating care in an intensive care unit.

## The detailed methodology for the UV-Net for 3D Lung Segmentation

## This algorithm, a cutting-edge deep learning approach for 3D lung segmentation, introduces a novel UV-Net composed of 2D encoder modules and 3D decoder modules, forming a U-shaped network structure that adapts to data characteristics (2). To enhance the model's receptive field and integrate global context information, an Atrous Spatial Pyramid Pooling (ASPP) module is incorporated, merging feature maps of varying scales to preserve both spatial and semantic information. Furthermore, during upsampling, the model employs a PixelShuffle technique to restore spatial resolution of feature maps, maintaining fine details and achieving a continuous and complete topology of pulmonary vessels and airways. The overall process involves initial segmentation of lung lobe, ensuring smooth and clear edges between different lobes. The airway model segments the tree-like structure of the lung's airways, maintaining their connectivity. Additionally, the vascular model segments a complete and connected vascular network. Ultimately, blood vessel volumes are calculated based on the cross-sectional area of each vessel. During internal validation, the algorithm demonstrated exceptional accuracy, achieving Dice coefficients of 92.68% for arteries and 89.96% for veins, underscoring its efficacy and precision in clinical applications.

## Comprehensive description of the AI system leveraging MVP-Net and 3D U-Net for pneumonia lesion detection and segmentation

This system was constructed upon deep convolutional neural networks and trained on a vast dataset comprising thousands of annotated CT images (3). To ensure the utmost accuracy, three pivotal modules were devised: pneumonia lesion detection, pneumonia lesion segmentation, and lung lobe segmentation. Initially, an MVP-Net was employed to identify bounding boxes enclosing pneumonia lesions, specifically targeting areas of consolidation and ground-glass opacities (GGOs). Exploiting a channel-wise attention mechanism and incorporating multiple inputs with varying window centers and widths, the system effectively captured the spatial context of pneumonia, thereby enhancing detection sensitivity. Furthermore, a symptom classifier is then used to differentiate between consolidation and ground-glass opacities. Subsequently, a 3D U-Net was employed to extract the pneumonia lesions, i.e., the voxels containing pneumonia. Lastly, an anatomical prior embedded network was trained to partition the extracted voxels into the five pulmonary lobes. Consequently, quantitative analysis could be computed for each pneumonia symptom, enabling a more comprehensive assessment.

This system exhibited robust performance in lung lobe and lesion segmentation, achieving Dice coefficients of 0.97 in the HOUSE dataset and 0.89 in the challenging LOLA dataset, validating its effectiveness across varied conditions. For lesion recognition, when evaluated on internal testing datasets it demonstrated high sensitivities of 90.1% and 90.3% for consolidation and ground-glass opacity detection, respectively, with low false positives rates of 1.8 and 1.4 per scan. The lesion segmentation accuracy was reliably quantified at a Dice coefficient of 0.82, underlining the system's precision and clinical applicability. Reference

1. National Health Commission of the People’s Republic of China. Guidelines for the diagnosis and treatment of COVID-19 (10th Trial Version). [Internet]. 2023 [cited 2023 Mar 13]. Available from: http://www.gov.cn/zhengce/zhengceku/2023-01/06/5735343/files/5844ce04246b431dbd322d8ba10afb48.pdf (in Chinese)

2. Bai Y, Wang X, Zhou Z, Wu Z, Feng Q, Qi J. Pulmonary Segments Segmentation with Hierarchical Weak Labels. 2023 IEEE 20th International Symposium on Biomedical Imaging (ISBI). 2023 Apr 18;1–5.

3. Xu Q, Zhan X, Zhou Z, Li Y, Xie P, Zhang S, et al. AI-based analysis of CT images for rapid triage of COVID-19 patients. NPJ Digit Med. 2021 Apr 22;4:75.

# Supplementary Tables

## Supplementary Table 1 CT findings on follow-up CT images

| CT features | Overall |
| --- | --- |
| Number of cases | 122 |
| Normal CT appearance | 20 (16.40) |
| GGO | 103 (84.43) |
| Consolidation | 15 (12.30) |
| Pleural thickening | 46 (37.70) |
| Pleural effusion | 21 (17.21) |
| Pericardial effusion | 17 (13.93) |
| Lymph node enlargement | 13 (10.66) |
| Emphysema | 26 (21.31) |
| Bronchial or bronchioles wall thickening | 49 (40.16) |
| Linear atelectasis | 61 (50.00) |
| Pleuroparenchymal band | 31 (25.41) |
| Bronchiectasis and/or bronchiolectasis | 25 (20.49) |
| Fibrotic interstitial lung abnormalities | 68 (55.74) |
| Traction bronchiectasis and/or bronchiolectasis | 36 (29.51) |
| Reticulation | 20 (16.39) |
| Architectural Distortion | 52 (42.62) |

Note: Except where indicated, the data are the numbers of patients, with percentages in parentheses.

Abbreviations: GGO: ground-glass opacity.

| Laboratory results | All cohort | Nonfibrotic cohort | Fibrotic cohort | *p* |
| --- | --- | --- | --- | --- |
| Number of cases | 122 | 54 | 68 | / |
| Patients with complete regular blood test | 114(93.44) | 48(88.89) | 66 (97.06) | / |
| Anemia | 40 (32.79) | 8 (14.81) | 32 (47.06) | 0.001* |
| WBC^‡^>9.5×10^9^ | 22 (18.03) | 6 (11.11) | 16 (23.53) | 0.20 |
| LY^‡^<0.8×10^9^ | 0 (0) | 0 (0) | 0 (0) | NA |
| NEUT^‡^>7.5×10^9^ | 113 (92.62) | 48 (88.89) | 65 (95.59) | 1.00 |
| EOS^‡^ < 0.02×10^9^ | 47 (38.52) | 18 (33.34) | 29 (42.65) | 0.62 |
| Thrombocytopenia (PLT^‡^<100×10^9^) | 23 (18.85) | 9 (16.67) | 14 (20.59) | 0.96 |
| hsCRP |  |  |  |  |
| hsCRP>50 mg/l | 48 (39.34) | 13 (24.07) | 35 (51.47) | 0.01* |
| Missing | 11(9.02) | 8(14.81) | 3(4.41) | / |

## Supplementary Table 2 Group comparison of initial laboratory characteristics

Note: Except where indicated, the data are the numbers of patients, with percentages in parentheses. Percentages and *p* values were calculated in cohort with complete test results. Abbreviations: WBC: white blood cell; NEUT: neutrophil; LY: lymphocyte; EOS: eosinophil; hs-CRP, high-sensitivity C-reactive-protein; NA, not applicable.

^‡^indicates the number of blood cells per liter of blood.

*denotes *p*<0.05.

## Supplementary Table 3 Group comparison of initial clinical symptoms

| Main clinical symptoms | All cohort | Nonfibrotic cohort | Fibrotic cohort | *p* |
| --- | --- | --- | --- | --- |
| Number of cases | 122 | 54 | 68 | / |
| Chest tight | 49 (40.16) | 15 (27.78) | 34 (50.00) | 0.02* |
| Cough | 85 (69.67) | 35 (64.81) | 50 (73.53) | 0.40 |
| Sputum | 71 (58.20) | 29 (53.70) | 42 (61.76) | 0.48 |
| Blood-stained sputum | 9 (7.38) | 3 (5.56) | 6 (8.82) | 0.74 |
| Rhinorrhea | 38 (31.15) | 13 (24.07) | 25 (36.76) | 0.19 |
| Sore throat | 46 (37.70) | 17 (31.48) | 29 (42.65) | 0.28 |
| Diarrhea | 6 (4.92) | 2 (3.70) | 4 (5.88) | 0.90 |
| Nausea | 19 (15.57) | 8 (14.81) | 11 (16.18) | 1.00 |
| Chest pain | 5 (4.10) | 3 (5.56) | 2 (2.94) | 0.79 |
| Drowsiness | 11 (9.02) | 2 (3.70) | 9 (13.24) | 0.13 |
| Apathy | 13 (10.66) | 3 (5.56) | 10 (14.71) | 0.18 |
| Chills | 24 (19.67) | 9 (16.67) | 15 (22.06) | 0.61 |
| Fatigue | 26 (21.31) | 13 (24.07) | 13 (19.12) | 0.66 |

Note: Except where indicated, the data are the numbers of patients, with percentages in parentheses.

*denotes *p*<0.05.

## Supplementary Table 4 Cut-Off values, AUC (95% CI), Accuracy, Sensitivity, Specificity, Precision, F1 score of CT Quantitative Parameters and CT score

| CT features | AUC (95% CI) | Accuracy | Sensitivity | Specificity | Precision | F1 score | Cutoff value |
| --- | --- | --- | --- | --- | --- | --- | --- |
| PV (ml) | 0.79(0.71-0.86) | 0.71 | 0.69 | 0.74 | 0.77 | 0.73 | 182.10 |
| CV (ml) | 0.80(0.72-0.88) | 0.73 | 0.75 | 0.70 | 0.76 | 0.76 | 39.58 |
| GV (ml) | 0.78(0.70-0.86) | 0.71 | 0.68 | 0.76 | 0.78 | 0.72 | 136.25 |
| CT score | 0.77(0.69-0.85) | 0.71 | 0.54 | 0.93 | 0.90 | 0.68 | 13.50 |
| PM (g) | 0.79(0.71-0.87) | 0.72 | 0.69 | 0.76 | 0.78 | 0.73 | 105.45 |
| BV5% | 0.65(0.55-0.75) | 0.64 | 0.69 | 0.57 | 0.40 | 0.51 | 42.73% |
| PA (HU) | 0.58(0.48-0.69) | 0.62 | 0.72 | 0.5 | 0.64 | 0.68 | -426.77 |

Abbreviations: AUC: area under curve; CI: confidence interval; PV: pneumonia volume; CV: consolidation volume; GV: ground-glass opacity volume; PM: pneumonia mass; BV5%: the percentage of the volume of blood contained in vessels with cross-sectional areas less than 5 mm^2^; PA: the attenuation of pneumonia.

## Supplementary Table 5 Univariate logistic regression analysis of demographic, clinical and laboratory findings.

| Characteristics | Odd ratio | 95%CI | *p* |
| --- | --- | --- | --- |
| Demographic characteristics | |  |  |
| Age > 68.50 years | 5.44 | 2.49-11.89 | <0.001* |
| Male | 0.48 | 0.23-1.00 | 0.0497* |
| Severe/critical clinical type | 5.92 | 2.56-13.68 | <0.001* |
| Laboratory findings |  |  |  |
| Anemia | 4.15 | 1.80-9.56 | <0.001* |
| WBC^‡^>9.5×10^9^ | 1.35 | 0.56-3.28 | 0.50 |
| EOS^‡^< 0.02×10^9^ | 1.24 | 0.60-2.57 | 0.56 |
| Thrombocytopenia (PLT^‡^<100×10^9^) | 1.01 | 0.42-2.46 | 0.98 |
| hsCRP>50 mg/L | 2.39 | 1.14-5.00 | 0.02* |

Abbreviations: WBC: white blood cell; NEUT: neutrophil; LY: lymphocyte; EOS: eosinophil; hsCRP: high-sensitivity C-reactive-protein.

Note: The median of age, 68.50 years old, was taken as the cutoff value of age.

^‡^indicates the number of blood cells per liter of blood.

*denotes *p*<0.05.

## Supplementary Table 6 Group comparison of demographic and clinical characteristics in the validation dataset.

| Characteristics | All cohort | Nonfibrotic cohort | Fibrotic cohort | *p* |
| --- | --- | --- | --- | --- |
| Number of cases | 45 | 19 | 26 | / |
| Sex |  |  |  | 1 |
| Male | 25 (55.56) | 11 (57.89) | 14 (53.85) | / |
| Female | 20 (44.44) | 8 (42.11) | 12 (46.15) | / |
| Age (mean (SD), years) | 67.29 (14.29) | 61.63(16.00) | 71.42 (11.52) | 0.02* |
| Clinical type |  |  |  | 0.06 |
| Mild/moderate | 20 (44.44) | 12 (63.16) | 8 (30.77) | / |
| Severe/critical | 25 (55.56) | 7 (36.84) | 18 (69.23) | / |
| Comorbidity | 36 (80.00) | 16 (84.21) | 20 (76.92) | 0.82 |
| Diabetes | 11 (24.44) | 7 (36.84) | 4 (15.38) | 0.19 |
| Cardiovascular  disease | 26 (57.78) | 11 (57.89) | 15 (57.69) | 1.00 |
| Cerebral artery disease | 3 (6.67) | 1 (5.26) | 2 (7.69) | 1.00 |
| Chronic lung disease | 3 (6.67) | 1 (5.26) | 2 (7.69) | 1.00 |
| Chronic kidney disease | 3 (6.67) | 0 (0.00) | 3 (11.54) | 0.35 |
| Chronic liver  disease | 1 (2.22) | 1 (5.26) | 0 (0.00) | 0.87 |
| Immunocompromised status or malignancies | 8 (17.78) | 5 (26.32) | 3 (11.54) | 0.38 |

Note: Except where indicated, the data are the numbers of patients, with percentages in parentheses. SD: Standard error.

* denotes *p*<0.05.

## Supplementary Table 7 Cut-Off values, AUC (95% CI), Accuracy, Sensitivity, Specificity, Precision, F1 score of CT Quantitative Parameters and CT score in the validation dataset.

| CT features | AUC (95% CI) | Accuracy | Sensitivity | Specificity | Precision | F1 score | Cutoff value |
| --- | --- | --- | --- | --- | --- | --- | --- |
| PV (ml) | 0.86 (0.76-0.97) | 0.80 | 0.77 | 0.84 | 0.87 | 0.82 | 270.87 |
| CV (ml) | 0.90 (0.82-0.99) | 0.84 | 0.88 | 0.79 | 0.85 | 0.87 | 41.79 |
| GV (ml) | 0.83 (0.71-0.95) | 0.78 | 0.73 | 0.84 | 0.86 | 0.79 | 227.64 |
| PM (g) | 0.88 (0.78-0.98) | 0.80 | 0.77 | 0.84 | 0.87 | 0.82 | 155.35 |
| BV5% | 0.68 (0.52-0.84) | 0.69 | 0.50 | 0.95 | 0.42 | 0.46 | 52.35% |
| PA (HU) | 0.83 (0.71-0.95) | 0.78 | 0.77 | 0.79 | 0.83 | 0.80 | -428.40 |
| CT score | 0.85 (0.73-0.97) | 0.82 | 0.92 | 0.68 | 0.80 | 0.86 | 11.00 |

Abbreviations: AUC: area under curve; CI: confidence interval; PV: pneumonia volume; CV: consolidation volume; GV: ground-glass opacity volume; PM: pneumonia mass; BV5%: the percentage of the volume of blood contained in vessels with cross-sectional areas less than 5 mm^2^; PA: the attenuation of pneumonia.

## Supplementary Table 8 Univariate logistic regression analysis of demographic, clinical and laboratory findings in the validation dataset.

| Characteristics | Odd ratio | 95%CI | *p* |
| --- | --- | --- | --- |
| Demographic characteristics | |  |  |
| Age > 69.00 years | 1.71 | 0.51-5.74 | 0.38 |
| Male | 1.18 | 0.36-3.89 | 0.79 |
| Severe/critical clinical type | 3.86 | 1.11-13.46 | 0.03* |
| Laboratory findings |  |  |  |
| Anemia | 1.38 | 0.34-5.62 | 0.65 |
| WBC^‡^>9.5×10^9^ | 6.22 | 1.45-26.64 | 0.01* |
| Neut^‡^>7.5×10^9^ | 11.59 | 2.21-60.9 | 0.01* |
| Lymph^‡^<0.8×10^9^ | 3.47 | 0.99-12.09 | 0.051 |
| EOS^‡^< 0.02×10^9^ | 2.44 | 0.70-8.52 | 0.16 |
| Thrombocytopenia (PLT^‡^<100×10^9^) | 2.35 | 0.22-24.51 | 0.48 |
| hsCRP>50 mg/L | 3.21 | 0.84-12.35 | 0.09 |

Abbreviations: WBC: white blood cell; NEUT: neutrophil; LY: lymphocyte; EOS: eosinophil; hsCRP: high-sensitivity C-reactive-protein.

Note: The median of age, 69.00 years old, was taken as the cutoff value of age.

^‡^indicates the number of blood cells per liter of blood.

*denotes *p*<0.05.

## Supplementary Table 9 Univariate and multivariate logistic regression analysis of CT Quantitative Parameters and CT score in the validation dataset.

|  | Unadjusted model (n=122) | | | Adjusted model (n=122) | | |
| --- | --- | --- | --- | --- | --- | --- |
| CT features | Odd ratio | 95%CI | *p* | Odd ratio | 95%CI | *p* |
| PV > cutoff | 17.78 | 3.83-82.43 | <0.001* | 19.09 | 3.05-221.42 | 0.005* |
| CV > cutoff | 28.75 | 5.62-147.06 | <0.001* | 42.78 | 5.36−979.68 | 0.002* |
| GV > cutoff | 14.48 | 3.21-65.35 | <0.001* | 15.29 | 2.64−147.58 | 0.006* |
| PM > cutoff | 17.78 | 3.83-82.43 | <0.001* | 19.09 | 3.05−221.42 | 0.005* |
| BV5% > cutoff | 0.06 | 0.01-0.48 | 0.009* | 0.06 | 0.003−0.46 | 0.02* |
| PA > cutoff | 12.50 | 2.99-52.31 | <0.001* | 9.54 | 2.13−53.41 | 0.005* |
| CT score > cutoff | 26.00 | 4.58-147.66 | <0.001* | 19.47 | 3.46−181.83 | 0.002* |

Note: Adjusted models used clinical type, and abnormal laboratory tests (higher white blood cell and neutrophil) as covariates. The median of age, 69.00 years, was used as the age cutoff value.

Abbreviations: PV: pneumonia volume; CV: consolidation volume; GV: ground-glass opacity volume; PM: pneumonia mass; BV5%: the percentage of the volume of blood contained in vessels with cross-sectional areas less than 5 mm^2^; PA: the attenuation of pneumonia.

* denotes *p*<0.05

Supplementary Figure

##
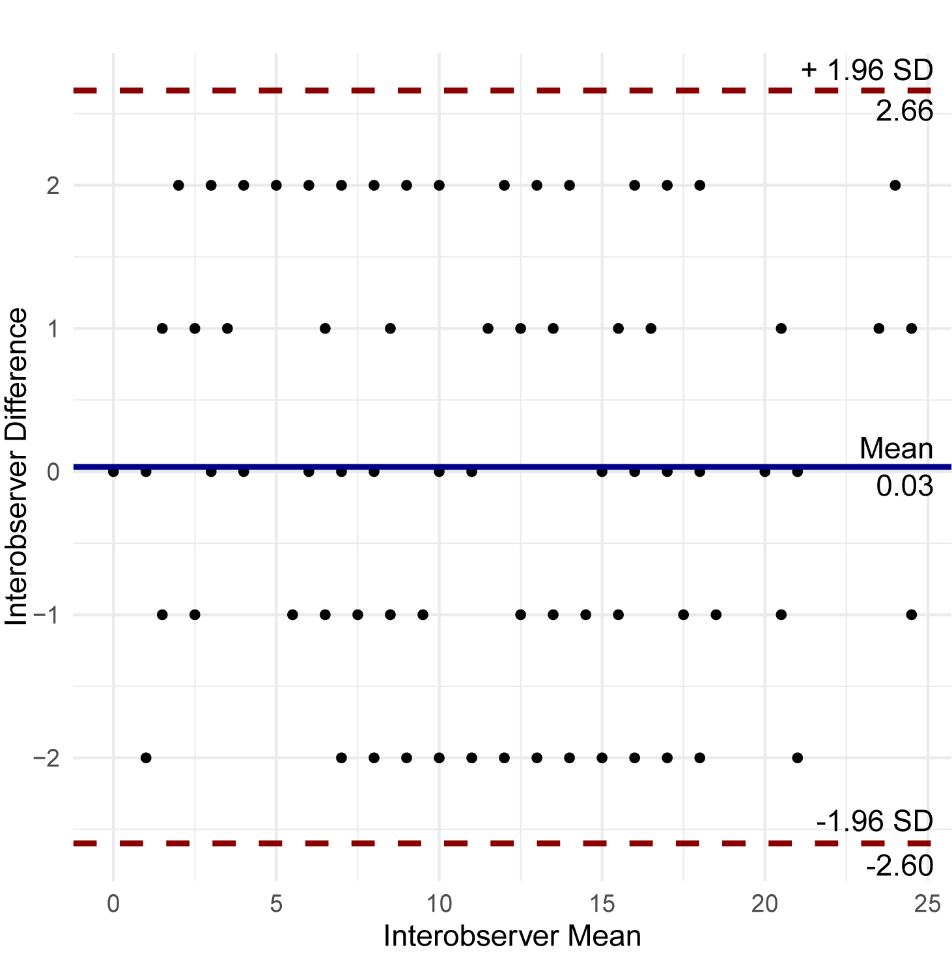


Supplementary Figure 1 Bland-Altman Plot Demonstrating Interobserver Agreement. The x-axis represents the average CT scores from two radiologists, while the y-axis shows their scoring differences. Each point illustrates the discrepancy for an individual patient. The central blue line indicates the mean difference (0.03), and the red dashed lines mark the 95% limits of agreement (±1.96 SD), demonstrating substantial agreement between two observers.
